# Effects of participation in a cross year peer tutoring programme in clinical examination skills on volunteer tutors' skills and attitudes towards teachers and teaching

**DOI:** 10.1186/1472-6920-7-20

**Published:** 2007-06-28

**Authors:** Sharon Buckley, Javier Zamora

**Affiliations:** 1 University of Birmingham, Medical School Education Unit, Vincent Drive, Edgbaston, Birmingham, B15 2TT, UK; 2 Clinical Biostatistics Unit, Hospital Ramon y Cajal, Complutense University of Madrid, 28034, Madrid

## Abstract

**Background:**

Development of students' teaching skills is increasingly recognised as an important component of UK undergraduate medical curricula and, in consequence, there is renewed interest in the potential benefits of cross-year peer tutoring. Whilst several studies have described the use of cross-year peer tutoring in undergraduate medical courses, its use in the clinical setting is less well reported, particularly the effects of peer tutoring on volunteer tutors' views of teachers and teaching. This study explored the effects of participation in a cross-year peer tutoring programme in clinical examination skills ('OSCE tutor') on volunteer tutors' own skills and on their attitudes towards teachers and teaching.

**Methods:**

Volunteer tutors were final year MBChB students who took part in the programme as part of a Student Selected Component (SSC). Tutees were year 3 MBChB students preparing for their end of year 'OSCE' examination. Pre and post participation questionnaires, including both Likert-type and open response questions, were used. Paired data was compared using the Wilcoxon signed-rank test. All tests were two-tailed with 5% significance level.

**Results:**

Tutors reflected their cohort in terms of gender but were drawn from among the more academically successful final year students. Most had previous teaching experience. They were influenced to participate in 'OSCE tutor' by a desire to improve their own teaching and associated generic skills and by contextual factors relating to the organisation or previous experience of the OSCE tutor programme. Issues relating to longer term career aspirations were less important. After the event, tutors felt that participation had enhanced their skills in various areas, including practical teaching skills, confidence in speaking to groups and communication skills; and that as a result of taking part, they were now more likely to undertake further teacher training and to make teaching a major part of their career. However, whilst a number of students reported that their views of teachers and teaching had changed as a result of participation, this did not translate into significant changes in responses to questions that explored their views of the roles and qualities required of a good clinical teacher.

**Conclusion:**

Findings affirm the benefits to volunteer tutors of cross-year peer tutoring, particularly in terms of skills enhancement and reinforcement of positive attitudes towards future teaching responsibilities, and have implications for the design and organisation of such programmes.

## Background

The UK General Medical Council (GMC) has recently highlighted the importance of including, within the undergraduate medical curriculum, opportunities for students to develop teaching skills. In particular, it has emphasised the need for students to understand the principles of education as applied to medicine, be familiar with a range of teaching and learning techniques, and recognise their obligation to teach colleagues [1]. Within this context, cross-year peer tutoring is uniquely placed to contribute to meeting these requirements and is increasingly finding a place in undergraduate medical education, with reported benefits to both tutors and tutees [2]. However, cross-year peer tutoring within the clinical setting is less well reported [3-5], particularly the potential effects of participation in such programmes on volunteer tutors' attitudes towards teaching as part of their future professional activity.

In 2001, a voluntary cross-year peer tutoring programme, 'OSCE tutor', was introduced to the undergraduate medical course at the University of Birmingham as an option within a six week Student Selected Component (SSC) in which final year students organise their own sequence of learning activities. The purpose of 'OSCE tutor' was three fold: to develop final year students' teaching skills (including associated generic skills of confidence in speaking to groups, communication etc) and to foster their interest in teaching; to provide year 3 students with additional practice in clinical examination prior to their end of year 'OSCE'; and, indirectly, to improve the clinical learning of final year students, on the principle that teaching a skill requires full understanding on the part of the tutor. Participation in 'OSCE tutor' by final year students increased from 10 in 2001 to more than 90 (approximately one third of the final year cohort) in 2004.

In 2004, preparations to introduce further teaching opportunities for undergraduate medical students began. As part of these, we explored the effects of participation in the existing cross-year peer tutoring programme on volunteer tutors' skills and attitudes.

## Methods

### Context and organisation of the OSCE tutor programme

The 2004–05 OSCE tutor programme was used for our study. Two students organised the programme, with the support of staff from the Medical School Education Unit (MSEU). Volunteer tutors were grouped into teams, each of which was responsible for planning and delivering small group revision sessions on the clinical examination of a particular body system. Planning began in February 2005 with a training session on the basics of small group teaching and a briefing on year 3 assessment requirements. Subsequently, MSEU staff met regularly with the student organisers to support and assist the development of the programme. Revision sessions were held in May 2005, in the week prior to the end of year 3 'OSCE' examination. Each lasted one hour, focused on the examination of a particular body system; and included the opportunity for participants to practice their skills on each other. On-line sign up records indicate that 271 out of 350 year 3 students took part.

As part of their SSC assessment, all final year students were required to submit a learning plan for their proposed activities and a report of what they had actually done during the six weeks. Volunteer OSCE tutors recorded their intentions to take part in OSCE tutor in their learning plan and whether they actually did take part in their final report.

### Questionnaires

Our study was based on a prospective method. Volunteer year 5 tutors completed questionnaires at the start of the programme (Feb 2005) and after they had planned and delivered their small group sessions (May 2005). Pre-OSCE tutor questionnaires were completed at the teacher training sessions held for volunteer tutors; post-OSCE tutor questionnaires were distributed and collected by the student organisers shortly after the actual revision sessions had taken place.

The pre-OSCE tutor questionnaire used questions with responses on Likert-type scales and open response questions to explore student motivations for taking part in OSCE tutor, their views of teachers and teaching, previous teaching experience and their longer term career aspirations. The post-OSCE tutor questionnaire explored the perceived benefits of taking part, and whether participation had increased the likelihood that they would undertake further teacher training. It also revisited tutor perceptions on the roles and qualities required for good clinical teaching and their perceptions of longer term career direction. Likert-type scales ranged from 1 (not at all important) to 6 (very important); or 1 (strongly disagree) to 6 (strongly agree) as appropriate. Both questionnaires included a voluntary section for respondents to complete their personal details, but anonymous replies were also accepted.

Volunteer tutor characteristics, including gender and academic attainment were compared to that of the whole cohort. All 247 final year students were ranked 1 (highest achieving) to 247 (lowest achieving) according to their average mark across the six disciplines that make up the final year programme. The position of each volunteer tutor within the ranking provided an estimate of that student's academic attainment in comparison to the cohort as a whole.

### Statistical methods

Pre- and post-OSCE tutor questionnaire responses are graphically described as percentages of responses within the 1 to 3 points interval or in the 4 to 6 points interval of the 6-point Likert-type scales. When comparing pre and post responses, the percentage of students whose point scores increased, decreased or remained unchanged are shown. As long as quantitative data measured with Likert-type scales showed skewed distributions, comparisons between paired data were done using the Wilcoxon signed-rank test. Paired comparisons were defined either between the pre and post questionnaire of identified individuals, or between sections of responses within one questionnaire. All statistical analyses were carried out using SPSS v.12.0 (SPSS Inc). All tests were two-tailed with 5% significance level.

### Analysis of free text responses

Pre-questionnaire open text responses to the request to 'outline the direction you anticipate your career will take over the next few years' were grouped according to type of career stated ie primary care, hospital medicine (all specialties) and don't know/not sure.

Post-questionnaire open text responses to the request to give details of how taking part in OSCE tutor had changed their views of teachers and teaching were grouped according to whether they related to one of the identified roles of a good clinical teacher [6], the personal qualities required [7], the perceived difficulty of teaching or 'other'.

### Ethical approval

Agreement to undertake the project was obtained via the normal medical school procedures for approval of research involving MBChB students. Each questionnaire included a covering letter to students that explained the purpose of the research, that participation was voluntary and requesting their involvement.

## Results

### The characteristics of volunteer OSCE tutors

105 year 5 students indicated their intention to act as OSCE tutors either by attending the teacher training session or on their SSC learning plan. Of these, 82 completed pre-questionnaires (response rate 78%). Post SSC reports indicated that 94 students actually taught on the OSCE tutor programme. Of these, 62 completed post-questionnaires (response rate 66%). Students who gave reasons for not pursuing an initial interest in OSCE tutor in their report tended to cite pressure of competing activities rather than lack of interest in teaching as the reason for their change of plan.

Of the 94 students who actually took part in the OSCE tutor programme, 46% (43) could be identified as having completed both pre- and post questionnaires, the remaining students choosing to submit one or both of their responses anonymously. All pre-post comparisons were made using the responses of this sub group.

68% (62) of volunteer tutors were female, compared with 60% (148) of the final year as a whole.

Median ranking of volunteer tutors with regard to overall academic achievement was 92 compared to 123 for the whole student cohort. Higher achieving students were more likely to have completed survey questionnaires (data not shown).

62% (51) pre-questionnaire respondents and 61% (38) post-questionnaire respondents reported previous teaching experience. 36% (30) and 29% (18) respectively had taught fellow students informally earlier in their undergraduate careers whilst and 15% (12) and 21% (13) had participated in sports or other tutoring in their community.

### The motivations of volunteer OSCE tutors

Given the many possible pair-wise comparisons between different motivating factors, pre-OSCE tutor questions relating to reasons for taking part in OSCE tutor were grouped into three areas: skills enhancement, context and career direction. To explore the relative importance to students of each of these, average Likert-type response scores between each area were compared.

**Figure 1:**
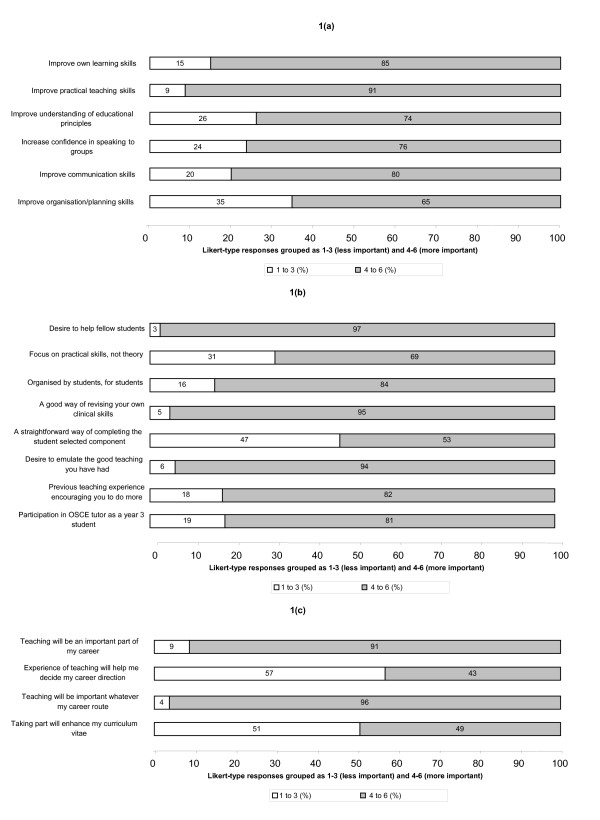
The motivations of volunteer tutors for joining the programme: analysis of pre-questionnaire responses (n = 82) to questions relating to skills enhancement, context of the 'OSCE tutor' programme and career direction. (a): motivations of volunteer tutors (skills enhancement). (b): motivations of volunteer tutors (context). (c): motivations of volunteer tutors (career direction)

Overall, responses to the pre-questionnaire indicated that many tutors joined the programme in order to improve their own educational or generic skills, including their practical teaching skills, confidence in speaking to groups, communication and own learning skills (Fig 1a). Contextual factors such as the opportunity to help fellow students, the desire to emulate the good teaching they had experienced and to revise their own clinical skills prior to entry to junior doctor training were also important (Fig 1(b)), with no statistical differences in Likert-type responses for these two areas (Wilcoxon p = 0.829).

Wider issues of career direction were less important than contextual factors (Wilcoxon p = 0.007) and than the desire for skills enhancement (Wilcoxon p = 0.002), with fewer students wishing to improve their curriculum vitae or further their decisions about their longer term career (Fig 1(c)). Whilst most pre-questionnaire respondents expected teaching to be an important part of any career route they followed, in supplementary open response questions, only 49% (40) described a clear career direction. Of these, 80% (32) wished to enter hospital medicine and the remainder primary care.

These findings held true for the group as a whole and when responses were analysed by gender, academic attainment, previous teaching experience or whether students were able to identify a clear career direction (data not shown).

### Effect of volunteering on tutors' perceptions of their skills and attitudes

**Figure 2:**
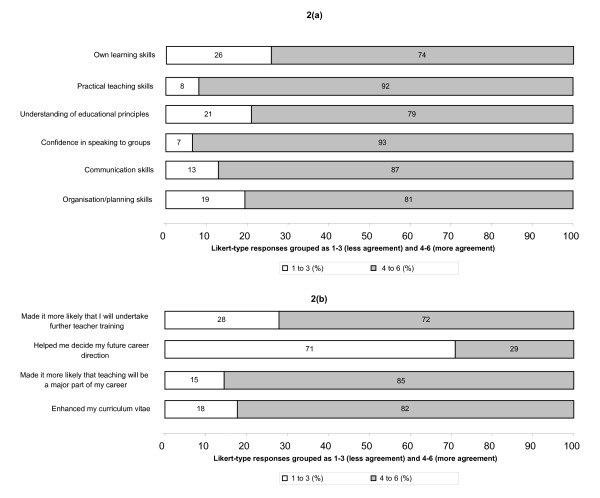
Volunteer tutors' views of the benefits of teaching on the OSCE tutor programme: analysis of post-questionnaire responses (n = 62) to questions relating to (a) skills enhancement and (b) career direction. (a): benefits to volunteer tutors (skills enhancement). (b): benefits to volunteer tutors (career direction)

Respondents to the post-OSCE tutor questionnaire reported that taking part in OSCE tutor had enhanced their skills in several areas (Fig 2(a)), including their practical teaching skills and their confidence in speaking to groups.

Furthermore, 85%(53) and 72%(45) respectively of post-questionnaire respondents indicated that OSCE tutor had increased the likelihood that teaching would be a major part of their career and that they would undertake further teacher training (Fig 2(b).

Overall, 82% of post-questionnaire respondents felt that taking part had enhanced their curriculum vitae. Within the 43 individuals who could be identified as having completed both pre- and post questionnaires, only 46% (20) indicated in the pre-questionnaire that enhancing their curriculum vitae was a motivating factor in joining OSCE tutor whereas 86% (37) agreed in the post-questionnaire that it had actually done *so*. However, in general, OSCE tutor did not help volunteer tutors decide their overall career direction (Fig 2(b)).

**Figure 3:**
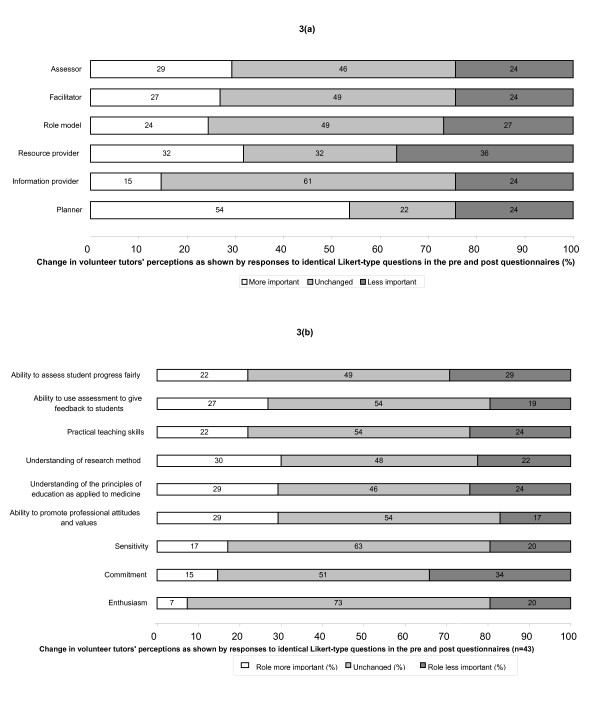
The effect of participation in the OSCE tutor programme on volunteer tutors' perceptions of the importance of (a) different teaching roles and (b) the personal qualities required of a good clinical teacher. (a): effects on volunteer tutors' perceptions (teaching roles). (b): effects on volunteer tutors' perceptions (personal qualities)

In answer to a 'yes/no' closed question, 40% (25) respondents to the post-questionnaire indicated that taking part in OSCE tutor had changed their views of teachers and teaching. Of these, 15 chose to elaborate with an open text comment (Table 1), with reference to the need for careful preparation and the perceived difficulty of teaching being the most common. However, analysis of the responses of the 43 individuals who had completed both before and after questionnaires did not show statistically significant differences in median scores for identical Likert-type questions that explored student views of either the perceived roles of a teacher [6] or the personal qualities required in a good clinical teacher [7] (Fig 3a and 3b). All Wilcoxon p values for this comparison were greater than 0.06.

**Table 1:** effect of participation in 'OSCE tutor' on volunteer tutors' views of teachers and teaching.

**Type of comment**	**Comment**
Teacher as planner	*Guess there should be a structure to it (teaching)*
	*Small group teaching much more effective when done properly with adequate preparation*
	*Idea of how important planning is in teaching*
	*Difficult to do well. Very easy to do moderately and very obvious when no preparation has been done at all*.
Teacher as facilitator	*Some teachers are poor at group interaction*
Enthusiasm/commitment of teacher	*(Being) passionate and genuinely keen to help helps to make teaching more effective*.
	*It's fun and could be done better*.
Teacher's sensitivity to the needs of students	*(Need to) understand the difficulties of difficult students*
Perceived difficulty of teaching	*Have started to question teaching skills and methods more closely*
	*It's harder than I thought*
	*It's nerve racking for the teachers*
	*It's harder than you anticipate*
	*As above, it's much more difficult than I thought*.
Miscellaneous	*Teachers have different styles, confidence, knowledge*.
	*Found it quite useful for myself and felt good about helping others, therefore would like to teach in the future eg anatomy tutor*

Responses of the 15 students who answered 'yes' to the question 'Has taking part in OSCE tutor changed your views of teachers and teaching?' and who chose to add additional comments are shown grouped according to relevance to the roles/qualities of a good teacher, perceived difficulty of teaching and 'miscellaneous' comments.

## Discussion

### Overview

This study offers insight into the characteristics and motivations of a group of final year medical students who voluntarily took part in a cross-year peer tutoring programme, and into the effects of participation on their perceptions of their skills and their attitudes towards teachers and teaching. It may also have implications for the design and delivery of teaching skills programmes for undergraduate medical students.

### Characteristics and motivations of tutors

Largely representative of their cohort in terms of gender, volunteer tutors tended to be drawn from among the academically higher achieving students within the year. A majority had previous teaching experience, either earlier informal teaching of their fellow students, or coaching or tutoring within their community. Motivations for participation included the desire to improve their skills (both practical teaching skills and associated generic skills such as confidence in speaking to groups) and contextual factors such as the desire to help fellow students. Although relatively few students identified a clear career direction and did not view developing their ideas in this regard as a reason for participating in OSCE tutor, a majority did see teaching as an important part of their future career.

### Skills benefits are associated with participation but tend to accrue to the more academic (and therefore less 'needy') students

Previous studies have shown that cross year peer tutoring programmes in medical education can enhance the skills of volunteer tutors and learners alike [2,4,8]. Our results affirm that peer tutoring can improve volunteer tutors' perceptions of their generic skills and illustrate that such benefits can occur in the clinical context. However, the preponderance of higher achieving final year students within the tutor cohort suggests that, where involvement in cross-year peer tutoring is voluntary, those students most in need of skills enhancement may not benefit. Some authors have recommended that tutors on cross-year peer tutoring programmes should be volunteers in order to allow less high achieving students to participate [9]. Our results suggest that such students may need encouragement to take advantage of the opportunities such programmes offer. This reflects the findings of similar studies in pre-clinical and clinical contexts [10].

### Re-inforcement of positive attitudes towards teaching and encouragement to further teacher training

Initially well-disposed to the idea of teaching, the majority of tutors reported that participation in OSCE tutor had increased their willingness to undertake further teacher training and to make teaching a major part of their professional practice. This suggests that cross-year peer tutoring can reinforce positive attitudes towards future teaching responsibilities, clearly a desirable outcome in the current climate of increased emphasis on the teaching role of clinical staff.

### Understanding of the teaching 'process' and reflection on the roles and qualities required of a good clinical teacher

Changes in volunteer tutors' perceptions of the complexities of the teaching process as a result of participation in 'OSCE tutor' were less marked, with relatively few reporting a change and even fewer identifying the nature of such a change. Whilst some students reported a greater appreciation of the need for planning and of the difficulty of teaching, responses to identical pre and post questions exploring the perceived importance of the varying roles and qualities required of a good clinical teacher showed no statistically significant differences. This may suggest that volunteer tutors' were relatively unreflective about the roles and qualities required of clinical teachers or that the changes brought about by participation in such a limited teaching programme were too small to be identified by Likert-type scales such as those used here. However, it also possible that experience of teaching may not, in itself, encourage students to consider the complexities of the teaching role and that, if this is required, it may be necessary to involve students in more structured opportunities for reflection as part of teaching skills programme.

### Effects on longer term career choices

Whilst 'OSCE tutor' was generally seen as having enhanced the curriculum vitae of a volunteer tutor, and fostered willingness to engage in teaching as part of their future practice, an influence on longer term career direction was not apparent. This may not be surprising in a context in which fewer than half of the volunteer tutors were either able or willing to articulate a clear career direction and with such a brief intervention as 'OSCE tutor'. However, it may also suggest that, alongside teaching skills programmes for undergraduates, raising awareness of medical career options is also important. It would be possible to combine both these needs by requiring students to shadow clinical teaching staff or to carry out a case study of the career paths of well-established clinical teachers.

### Limitations of the study and avenues for future research

#### Population

This study has only explored the views of volunteer tutors, a self-selected group that one might expect to be well-disposed towards teaching and which was biased towards higher achieving students. In future studies, it may be useful to compare the attitudes of volunteer tutors with those of the rest of the cohort to obtain a more comprehensive picture of student attitudes as a whole.

#### Response rates

In addition, the insights offered by this study are limited by the fact that the whilst the volunteer tutor group involved was relatively stable, and response rates to questionnaires within acceptable limits, pre- and post- OSCE tutor questionnaires could only be matched for approximately half of respondents. The opportunity to respond anonymously was allowed in order to encourage honest reporting, but this must be balanced against the reduction in potential for accurate pre and post comparisons. Similarly, voluntary completion of questionnaires allows the possibility that the results may be affected by differences in the views and perceptions of responders and non-responders. For example, it may be that responders are more reflective and analytical than the norm.

#### Methods used

Given the exploratory nature of our study, the statistical significances observed should be interpreted with caution and the hypotheses raised by analysis of the data confirmed in further studies specifically designed for the purpose. Furthermore, given that paired (pre-post) designs are powerful enough to detect statistical significance even for slight changes in scoring, the interpretation of these findings should take into account not only statistical significance but also the magnitude of the differences and these are, in the vast majority of the cases, slight. In such a context in which changes of view are relatively small, complementing questionnaires with more in depth qualitative studies such as group interviews or focus groups may provide greater insights than provided by Likert-type responses and limited free text questions alone. For example, they may allow greater exploration of changes in tutors' understanding of the teaching process, which may or may not be reflected in changes in opinion measurable on a Likert-type scale.

## Conclusion

Volunteer OSCE tutors were drawn from among the higher achieving students within the final year cohort and generally had some previous teaching experience. Relatively few articulated a clear career direction but most saw teaching as an important part of their future practice. Motivations for taking part in the programme included a desire to improve skills and the desire to help other students, with longer term career considerations being less important. Benefits of being an OSCE tutor included perceived improvement in teaching and associated generic skills, increased likelihood of engaging in teaching/teacher training in the future and enhancement of the curriculum vitae of participants. However, effects on tutors' views of teachers and teaching and on longer term career direction were less apparent. These findings affirm the benefits to volunteer tutors of cross-year peer tutoring within the undergraduate medical curriculum and have implications for the design and organisation of such programmes.

## Competing interests

We, the authors, declare that we have no competing interests that would influence this study.

## Authors' contributions

SB conceived of the study, participated in its implementation and jointly drafted the manuscript with JZ.

JZ analysed the data, prepared the figures and jointly prepared the manuscript with SB.

Both authors read and approved the final manuscript.

## Pre-publication history

The pre-publication history for this paper can be accessed here:


